# Leptin interferes with 3',5'-Cyclic Adenosine Monophosphate (cAMP) signaling to inhibit steroidogenesis in human granulosa cells

**DOI:** 10.1186/1477-7827-7-115

**Published:** 2009-10-22

**Authors:** Qing Lin, Song Ling Poon, Junling Chen, Linan Cheng, Basil HoYuen, Peter CK Leung

**Affiliations:** 1 Department of Obstetrics and Gynecology, Child and Family Research Institute, University of British Columbia, Vancouver, British Columbia, V6H 3V, Canada; 2 Department of Obstetrics and Gynecology, Beijing Friendship Hospital-Affiliate of Capital University of Medical Sciences, Beijing, PR China; 3 School of Medicine, Shanghai Jiao Tong University, Shanghai, 200030, PR China

## Abstract

**Background:**

Obesity has been linked to an increased risk of female infertility. Leptin, an adipocytokine which is elevated during obesity, may influence gonadal function through modulating steroidogenesis in granulosa cells.

**Methods:**

The effect of leptin on progesterone production in simian virus 40 immortalized granulosa (SVOG) cells was examined by Enzyme linked immunosorbent assay (ELISA). The effect of leptin on the expression of the steroidogenic enzymes (StAR, P450scc, 3betaHSD) in SVOG cells was examined by real-time PCR and Western blotting. The mRNA expression of leptin receptor isoforms in SVOG cells were examined by using PCR. SVOG cells were co-treated with leptin and specific pharmacological inhibitors to identify the signaling pathways involved in leptin-reduced progesterone production. Silencing RNA against leptin receptor was used to determine that the inhibition of leptin on cAMP-induced steroidogenesis acts in a leptin receptor-dependent manner.

**Results and Conclusion:**

In the present study, we investigated the cellular mechanisms underlying leptin-regulated steroidogenesis in human granulosa cells. We show that leptin inhibits 8-bromo cAMP-stimulated progesterone production in a concentration-dependent manner. Furthermore, we show that leptin inhibits expression of the cAMP-stimulated steroidogenic acute regulatory (StAR) protein, the rate limiting de novo protein in progesterone synthesis. Leptin induces the activation of ERK1/2, p38 and JNK but only the ERK1/2 (PD98059) and p38 (SB203580) inhibitors attenuate the leptin-induced inhibition of cAMP-stimulated StAR protein expression and progesterone production. These data suggest that the leptin-induced MAPK signal transduction pathway interferes with cAMP/PKA-stimulated steroidogenesis in human granulosa cells. Moreover, siRNA mediated knock-down of the endogenous leptin receptor attenuates the effect of leptin on cAMP-induced StAR protein expression and progesterone production, suggesting that the effect of leptin on steroidogenesis in granulosa cells is receptor dependent. In summary, leptin acts through the MAPK pathway to downregulate cAMP-induced StAR protein expression and progesterone production in immortalized human granulosa cells. These results suggest a possible mechanism by which gonadal steroidogenesis could be suppressed in obese women.

## Background

The major function of the female gonad is to differentiate and release mature oocytes for fertilization and successful propagation of the species. The follicular maturation, ovulation and corpus luteum function of mammalian ovaries is regulated by the interaction between gonadotropin releasing hormone, gonadotropins, ovarian sex steroids and a variety of peptide hormones [1]. Dysfunction in the biosynthesis of sex steroid hormones could impair normal ovarian function or even cause infertility.

Leptin, a key hormone in energy homeostasis and neuroendocrine function, has a permissive role in initiating puberty and is crucial in the pathogenesis of reproductive dysfunction in several disease states of energy imbalance [2]. In obesity, there is an expansion of the adipose tissue with increased leptin production [3]. Accumulating evidence suggests that increased leptin levels contribute to the pathogenesis of reproductive abnormalities including infertility, polycystic ovary syndrome (PCOS), anovulation, disruption of the menstrual cycle, hyperinsulinemia and many other conditions [4-6].

Recent studies that have identified expression of the leptin receptor in several peripheral tissues (eg. ovary, testis and adrenal gland) strongly suggest that leptin may have a direct effect on downstream endocrine targets of the reproductive axis [7,8]. *In vitro *studies conducted on thecal and granulosa cells have shown that leptin has negative effects on ovarian steroid output in rodent, bovine and human models. In particular, it has been found that: 1) Leptin antagonizes insulin action in human granulosa cells and thereby inhibits their gonadotropin-stimulated progesterone production [9]; 2) Leptin stimulates the release of proinflamatory cytokines and prostaglandins in human placenta [4]; 3) High serum and follicular fluid leptin may account for decreased fertilization, implantation and pregnancy rates of IVF in PCOS women [10]. Conditions with abnormally elevated leptin concentrations and impaired ovarian function may be due to disturbed steroidogenesis. However, the mechanisms by which leptin modulates steroidogenesis in the ovary remain elusive.

In the present study, we investigated the underlying mechanisms through which leptin modulates steroidogenesis in human granulosa cells. We found that treatment of immortalized granulosa cells with increasing doses of leptin inhibited 8-bromo cAMP-induced steroidogenesis with downregulation of the *de novo *produced steroidogenic acute regulatory (StAR) protein. In addition, we demonstrated that cAMP-regulated steroidogenic enzymes and progesterone production could be inhibited by leptin via the MAPK pathway.

## Methods

### Cell culture and chemicals

Studies of human ovarian granulosa cells have been limited by the small numbers and short life span in culture of cells currently obtained from clinical material. Using SV40 large T antigen, we have reproducibly immortalized freshly explanted human granulosa cells obtained through an in vitro fertilization program. The use of immortalized granulosa cells for this study was approved by the University of British Columbia Research Ethics Board. This cell line was shown to posses the same steroidogenic capabilities as primary human granulosa cells [11]. Cells are maintained in growth medium (M199:MCDB 105 (Sigma-Aldrich Corp., St. Louis, MO) containing 10% (v/v) fetal bovine serum (FBS; Hyclone Laborataries Inc., Logan, UT), 100 units/mL penicillin, 100 μg/mL streptomycin (Sigma) under a humidified atmosphere of 5% CO_2 _at 37°C and changed every 3 days. For each experiment, 2 × 10^5 ^immortalized granulosa cells were incubated in serum free medium for 4 h prior to treatment. Human recombinant leptin, 8-bromo cAMP, and SB203580 were purchased from Sigma. PD98059 and SP600125 were purchased from Calbiochem (San Diego, CA).

### RNA extraction and semiquantitave PCR

Total RNA was extracted with Trizol reagent (Invitrogen Inc., Burlington, ON, Canada) according to the manufacturer's instructions. Reverse transcription was performed in a mixture containing 5 μM random hexamer, 200 μM dNTP, 2 U/μl MMLV trasnscriptase (Promega, Madison, WI) and 1 μg RNA with the corresponding buffer at 42°C for 90 min followed by 85°C for 10 min. The cDNA was further amplified by specific primer pairs, including forward long-form leptin receptor (OBRb) (5'-CCA GAA ACG TTT GAG CAT CT-3') and reverse OBRb (5'-CAA AAG CAC ACC ACT CTC TC-3'), forward short-form leptin receptor (OBRa) (5'-GAA GGA GTG GGA AAA CCA AAG-3') and reverse OBRa (5'-CCA CCA TAT GTT AAC TCT CAG-3'), forward GAPDH (5'-TCC CAT CAC CAT CTT CCA-3') and reverse GAPDH (5'-CAT CAC GCC ACA GTT TCC-3').

### Real-time PCR

The primers used for SYBR Green real-time RT-PCR were designed using the Primer Express Software v2.0 (Applied Biosystems, Foster City, CA). The specific primer pairs used are forward StAR (5'-AAACTTACGTGGCTACTCAGCATC-3') and reverse StAR (5'-GACCTGGTTGATGATGCTCTTG-3'), forward P450scc (5'-CAGGAGGGGTGGACACGAC-3') and reverse P450scc (5'-AGGTTGCGTGCCATCTCATAC-3'), forward 3β-HSD (5'-GCCTTCAGACCAGAATTGAGAGA-3') and reverse 3β-HSD (5'-TCCTTCAAGTACAGTCAGCTTGGT-3'), forward GAPDH (5'-ATGGAAATCCCATCACCATCTT-3') and reverse GAPDH (5'-CGCCCCACTTGATTTTGG-3'). Real-time PCR was performed using the ABI prism 7000 Sequence 10 Detection System (Applied Biosystems) equipped with a 96-well optical reaction plate. The reactions were set up with 16.5 μl SYBRR Green PCR Master Mix (Applied Biosystems). All real-time experiments were run in triplicate and a mean value was used for the determination of mRNA levels. Negative controls, containing water instead of sample cDNA, were used in each experiment. Relative quantification of the mRNA levels for StAR, P450scc and 3β-HSD2 in ovarian cancer cells was performed using the comparative CT method with GAPDH as an internal standard and with the formula 2^-ΔΔCt^.

### Enzyme-Linked Immunosorbent Assay (ELISA)

Immunoplates were pre-coated with IgG (Calbiochem) and nonspecific binding sites were blocked with 0.1% (w/v) BSA buffer. IgG binds to the Fc fragment of the progesterone antibody while the Fab fragment is competitively bound by both biotinylated progesterone (EastCoast Bio, Inc. North Berwick, ME) and progesterone in the samples. The biotinylated progesterone interacts with streptavidin-horseradish peroxidase (SA-HRP) (EastCoast Bio), which catalyzes the substrate solution composed of 3,3',5,5'-tetramethylbenzidine (TMB) (Dojindo Inc., Gaithersburg, MD), to produce a blue-colored solution. The enzyme-substrate reaction is stopped by the addition of sulphuric acid (H_2_SO_4_) and the solution turns yellow. The intensity of the yellow color is directly proportional to the amount of biotinylated peptide-SA-HRP complex but inversely proportional to the amount of the progesterone in the samples. A standard curve of progesterone with known concentrations was established accordingly, and the progesterone concentrations in the samples were determined by extrapolation to this standard curve. The intra- and inter-assay CV of the progesterone assay were 5.6 and 10.2% respectively.

### Western blotting

The cells were washed twice with ice-cold PBS and lysed in RIPA buffer (150 mM NaCl, 50 mM Tris-base (pH 7.5), 1% (v/v) Nonidet P-40, 0.5% (w/v) sodium deoxycholate, and 0.1% (v/v) SDS). Twenty micrograms of total protein were run on 12% SDS-PAGE gels and electrotransferred to a nitrocellulose membrane (Biorad laboratories, Hercules, CA). The membrane was immunoblotted using specific primary antibodies (StAR, P450scc, 3β-HSD and β-actin: Santa Cruz Biotechnology Inc., Santa Cruz, CA; ERK, phosphorylated ERK, JNK, phosphorylated JNK and p38: Cell Signaling Technology Inc., Danvers, MA; phosphorylated p38: Biosource, Invitrogen) at 4°C overnight. After washing, the membranes were incubated with horseradish peroxidase-conjugated secondary antibody for 1 h, and visualized using the ECL system (GE Healthcare Bio-Science, Piscataway NJ).

### Statistical analysis

**Figure 1:**
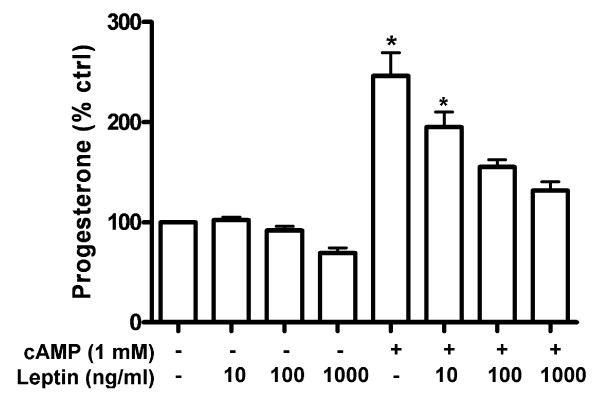
**Leptin inhibited 8-bromo cAMP-stimulated progesterone production in human granulosa cells**. Cells were treated with increasing concentrations of leptin (10 ng/ml - 1000 ng/ml) in the presence or absence of 1 mM 8-bromo cAMP for 24 h. Media were collected and assayed for progesterone production by ELISA. Each data point in the figure represents the mean ± SEM of three independent experiments. * above the column indicates that those groups differ significantly from untreated control (*P *< 0.05); (ctrl = control).

**Figure 2:**
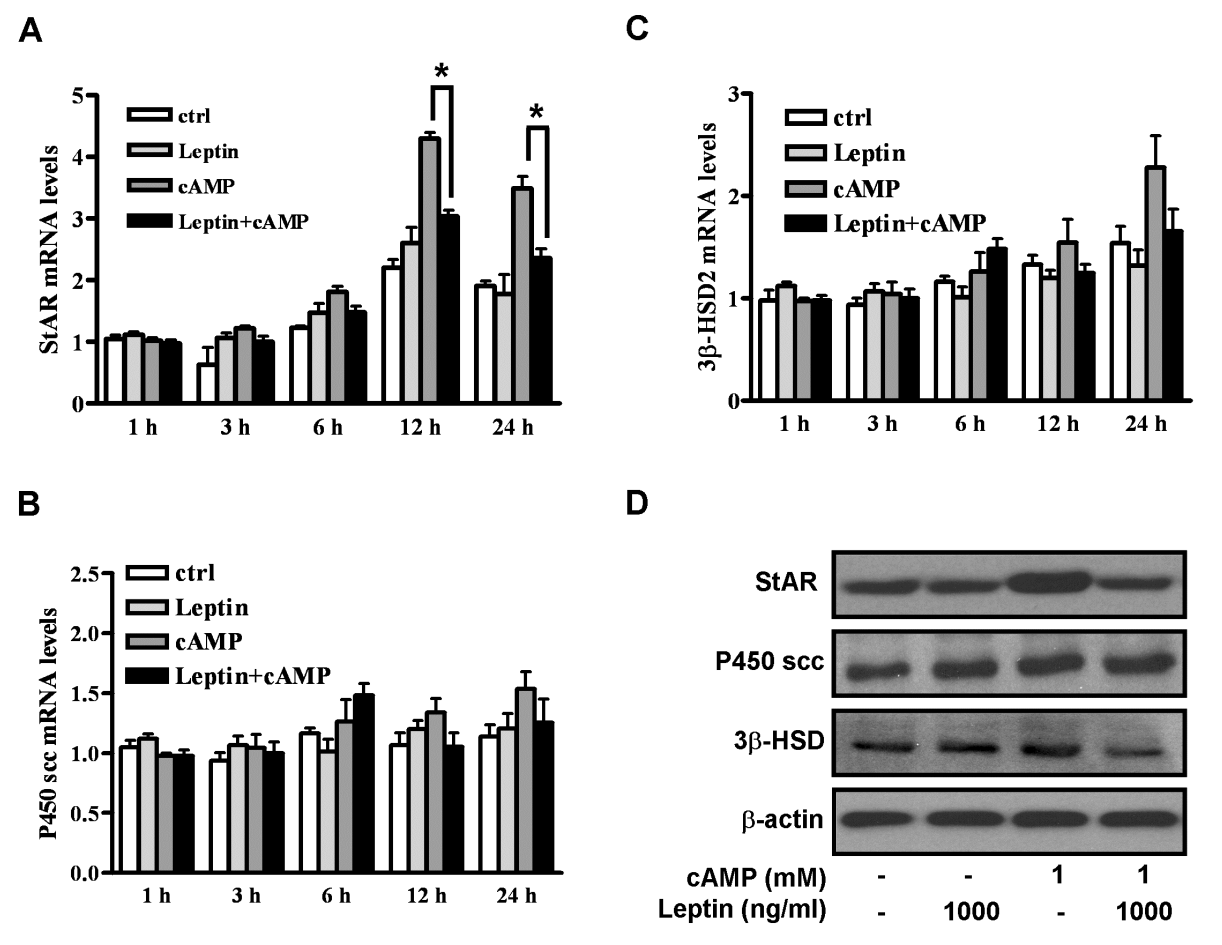
**Leptin inhibited 8-bromo cAMP-stimulated StAR mRNA and protein expression**. Cells were treated with leptin (1000 ng/ml) in the presence or absence of 8-bromo cAMP (1 mM) for different time scale (1, 3, 6, 12, 24 h) and total RNA was analyzed with real time RT-PCR to measure the temporal change of StAR mRNA (A), P450scc mRNA (B) and 3β-HSD mRNA (C) levels with GAPDH as internal standard. Cell lysates from cotreatment of leptin with 8-bromo cAMP for 24 h was subjected to Western blot analysis to monitor the protein levels of StAR, P450scc and 3β-HSD (D). The blots are a Western blotting representative data from three independent experiments. Each data point in the figure represents the mean ± SEM of three independent experiments. * above the bars in "A" indicate that cotreatment of leptin with cAMP group significantly differ from cAMP treated group in the particular time scale (*P *< 0.05). (ctrl = control).

**Figure 3:**
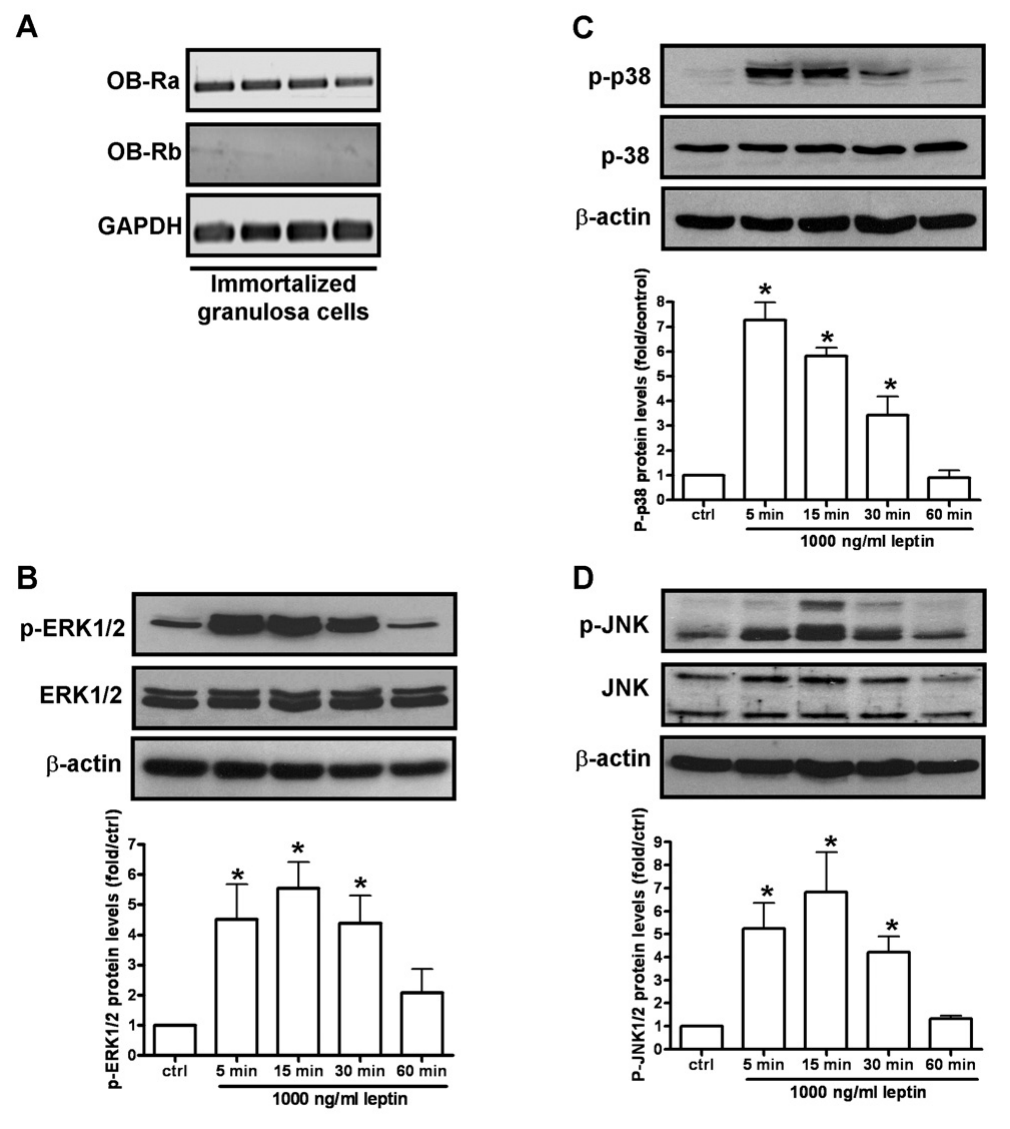
**Leptin induced MAPK pathway in human granulosa cells**. (A) The expression of leptin receptor in human immortalized granulosa cells were monitored by semi-quantitative RT-PCR. Upper bands showed the expression of short form leptin receptor (Ob-Ra) in 4 different passages of immortalized granulosa cells. Lower band is to detect the expression of long form leptin receptor (Ob-Rb) in immortalized granulosa cells. GAPDH was used as internal standard. Cells were treated with leptin (1000 ng/ml) for different time scale (5, 15, 30, 60 min) and cell lysates were collected and subjected to Western blot analysis to monitor the expression of phosphorylated ERK1/2 (B), phosphorylated p38 (C) and phosphorylated JNK (D). Total ERK1/2, p38, JNK and β-actin were detected as internal standard. Upper bands are Western blotting representative datas from three independent experiments. Lower bars were integrated optical density (IOD) of target phosphorylated protein expression with target total protein normalization. Each data point in the figure represents the mean ± SD of three independent experiments. * above the bars indicates that those groups differ significantly from untreated control (*P *< 0.05). (ctrl = control).

**Figure 4:**
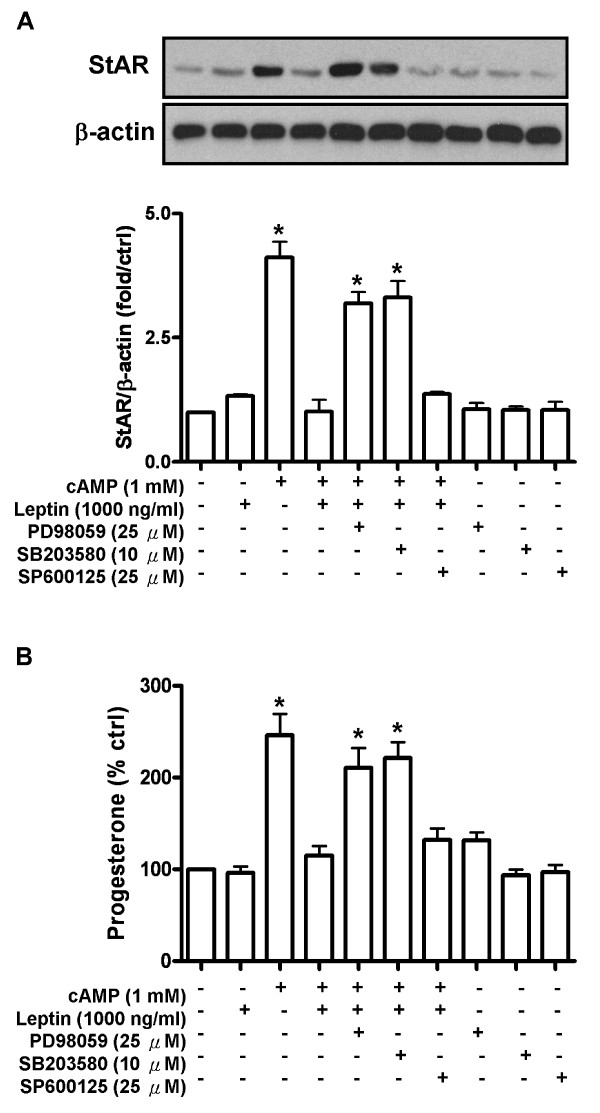
**Leptin acted through ERK1/2 and p-38 pathways to modulate steroidogenesis in human granulosa cells**. Cells were treated with leptin (1000 ng/ml) in the presence or absence of ERK1/2 inhibitor (PD98059, 25 μM), p38 inhibitor (SB203580, 10 μM) and JNK inhibitor (SP600125, 25 μM) for 24 h. Cell lysates were collected and subjected to Western blot analysis to monitor the expression of StAR protein (A) and media were collected and assayed for progesterone production by ELISA (B). Upper bands in (A) are Western blotting representative data from three independent experiments. Lower bars were integrated optical density (IOD) of StAR protein expression with β-actin normalization. Each data point in (A) represents the mean ± SD of three independent experiments. Each data point in (B) represents the mean ± SEM of three independent experiments. * above the bars indicate that those groups differ significantly from untreated control (*P *< 0.05).

**Figure 5:**
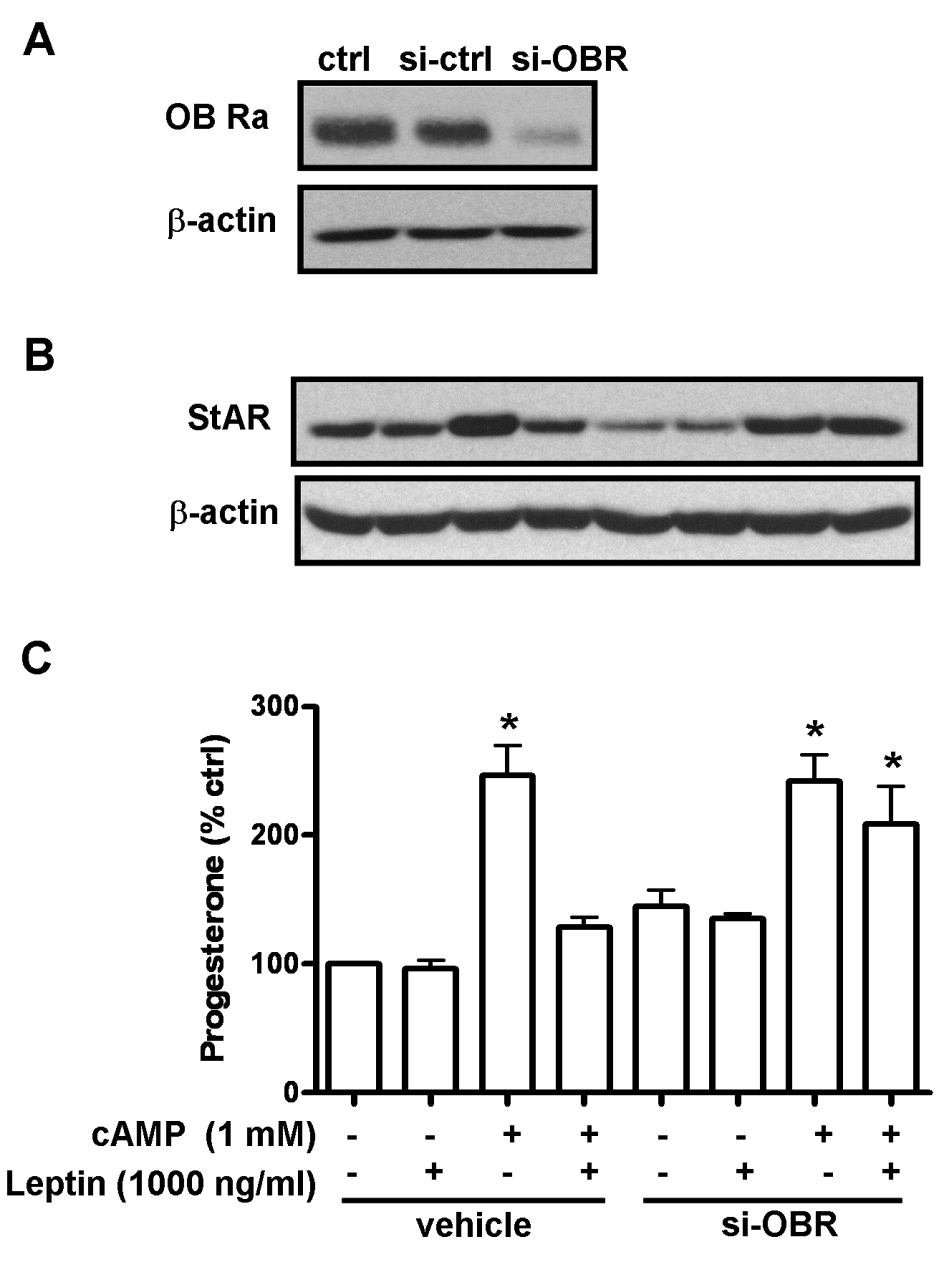
**Leptin acted through short form leptin receptor to downregulate the 8-bromo cAMP-stimulated steroidogenesis in human granulosa cells**. Cells were transfected with leptin receptor siRNA (si-OBR) for 24 h to reduce the endogenous expression of leptin receptor in human granulosa cells. Western blot analysis was used to monitor the efficiency of the siRNA (A). Transfected cells were then treated with leptin (1000 ng/ml) in the presence or absence of 8-bromo cAMP (1 mM) for 24 h and cell lysates were collected to detect the expressions of StAR protein (B) and media were collected and assayed for progesterone production (C). Blots in (B) are Western blotting representative data from three independent experiments. β-actin was detected as internal standard. Each data point in (C) represents the mean ± SEM of three independent experiments. * above the bars indicate that those groups differ significantly from untreated control in either vehicle or si-OBR group respectively (*P *< 0.05).

ELISA and real-time PCR normalized data are represented as mean ± S.E.M. of three independent experiments. Western blot normalized data are represented as mean ± S.D. of three independent experiments. Statistically significant differences between treatments and controls were determined by either two-way ANOVA followed by Bonferroni test (Figure 1 and Figure 2) or one-way ANOVA followed by Tukey test (Figure 3, 4, 5). Statistical significance was set at *P *< 0.05.

## Results

### Leptin attenuates the 8-bromo cAMP-stimulated progesterone production in human granulosa cells

The gonadotropin-stimulated progesterone production in granulosa and luteal cell is critical for normal uterine function, establishment and maintenance of pregnancy, and mammary gland development [1,12]. To gain insight into the impact of leptin on gonadotropin-stimulated steroidogenesis, we co-treated 8-bromo cAMP with increasing concentration of leptin (10 ng/ml, 100 ng/ml, 1000 ng/ml) for 24 h in immortalized human granulosa cells. Results showed that leptin inhibited the 8-bromo cAMP-stimulated progesterone production in a concentration-dependant manner, suggesting that leptin interferes with gonadotropin-stimulated progesterone production in these cells (Figure 1).

### Leptin inhibits the 8-bromo cAMP-induced StAR mRNA and protein induction in granulosa cells

The regulation of StAR, P450scc enzyme and 3β-HSD is crucial for regulating steroid hormone production in steroidogenic cells [13,14]. Using real-time PCR, we monitored the effect of leptin on these steroidogenic enzymes. Results showed that leptin inhibited the transcriptional mRNA level of StAR (Figure 2A), but not P450scc (Figure 2B) or 3β-HSD (Figure 2C), in a temporally-defined manner. In concordance with the mRNA regulation, the protein level of StAR induced by 8-bromo cAMP is significantly inhibited by the administration of leptin after 24 h of treatment (Figure 2D).

### Activation of ERK1/2 and p38 are involved in the leptin regulated 8-bromo cAMP-stimulated StAR protein expression and progesterone production

As we have previously shown, immortalized human granulosa cells only expressed the short form leptin receptor (Ob-Ra) (Figure 3A) [15]. It is documented that the MAPK pathway is the dominant signaling pathway downstream of Ob-Ra [16,17]. Thus, leptin treatment of human granulosa cells over different time intervals was used to monitor the activation of the MAPK pathway. As shown in our results, leptin induced the phosphorylation of ERK1/2 (Figure 3B), p38 (Figure 3C) and JNK (Figure 3D) in a time-dependent manner. To further elucidate the signaling pathways involved in the leptin-inhibited steroidogenesis in human granulosa cells, we used pharmacological inhibitors to individually block the ERK1/2, p38 and JNK pathways. The effectiveness of each inhibitor was tested prior to use in the current experiments (data not shown). Results showed that pretreatment for 30 minutes with the ERK1/2 inhibitor PD98059 or the p38 inhibitor SB203580 prior to co-treatment with 8-bromo cAMP and leptin reversed the inhibition of leptin on 8-bromo cAMP-induced StAR protein expression (Figure 4A) and progesterone production (Figure 4B) in granulosa cells. Interestingly, pretreatment with the JNK inhibitor SP600125 did not inhibit the leptin effect. These results suggest that the ERK1/2 and p38 MAPK pathways are necessary for leptin-mediated interference of cAMP-stimulated steroidogenesis in human granulosa cells.

### Leptin modulates steroidogenesis in human granulosa cells through the short form leptin receptor (OB-Ra)

To further confirm the role of the leptin receptor in leptin-modulated steroidogenesis in human granulosa cells, we used siRNA technology to knockdown the endogenous expression of the leptin receptor in these cells. The effectiveness of the siRNA was confirmed by Western blot analysis (Figure 5A). Knockdown of the leptin receptor in granulosa cells impaired the ability of leptin to inhibit 8-bromo cAMP-stimulated StAR protein expression (Figure 5B) and progesterone production (Figure 5C). These results confirm that leptin acts through its receptor on immortalized human granulosa cells to initiate the MAPK pathway and downregulate the cAMP-induced steroidogenesis.

## Discussion

Compelling evidence demonstrates a direct inhibitory action of leptin on steroid hormone secretion. Such effects have been independently reported by different groups in the three major steroidogenic tissues, namely the adrenal gland, the ovary and the testis [18-22]. However, the mechanisms for such an inhibitory action are only partially characterized, and little attention has been paid to the molecular events involved in leptin-induced inhibition of progesterone secretion in granulosa cells. To gain insight into the mechanisms whereby leptin suppresses progesterone secretion *in vitro*, we correlated the hormonal secretory responses to mRNA expression levels of StAR, P450scc and 3β-HSD in cAMP-stimulated immortalized human granulosa cells after exposure to high dosage of recombinant human leptin. In the present study we demonstrate that the physiological induction of StAR protein by cAMP, a rate-limiting step in steroidogenesis in steroidogenic cells, is significantly reduced by leptin treatment. These results confirm the predominantly inhibitory effects that leptin exerts on progesterone production in human granulosa cells. In addition, it was the first demonstration that the leptin short form receptor was involved in leptin-mediated inhibition of cAMP-stimulated steroidogenesis via the activation of the MAPK signaling pathway.

Leptin was shown to be distributed in the intact ovary and have distinct changes in its localization during folliculogenesis [23]. Study suggested that leptin may be produced locally and act in autocrine or paracrine way to affect steroidogenesis in the human ovary [23]. During obesity, both serum and follicular fluid levels of leptin may be as high as 100 ng/ml [24,25]and our study provides evidence that this high concentration of leptin suppressed the production of progesterone in granulosa cells. More importantly, treatment with a 1000 ng/ml of high dosage leptin also strikingly inhibited the cAMP-induced steroidogenesis in granulosa cells. This is in agreement with findings in PCOS demonstrating conspicuous occurrence of leptin in the wall of polycystic follicles, higher local concentration of leptin independent of the serum level with hormonal dysregulation in granulosa cells [26] which may account for the decreased pregnancy rate in these PCOS women [10].

Several reports have now demonstrated expression of biologically active isoforms of the leptin receptor in the endocrine pancreas [27], the ovary [8] and the placenta [28]. Consistent with this widespread expression, leptin can directly modulate the activity of these glands. Leptin has been shown to inhibit insulin secretion from pancreatic β-cells [29]. Leptin decreases the production of estradiol and progesterone from ovarian granulosa cells [30,31], at least partly via inhibition of the electron transport protein adrenodoxin [31]. Finally, leptin can modulate the release of hCG from human trophoblast cells in culture [32]. In our cell model, it was shown that only the leptin short form (Ob-Ra) receptor is expressed in the culture system. Interestingly, using siRNA to knockdown the endogenous leptin receptor reversed the inhibition of leptin on cAMP-induced StAR protein expression and progesterone production. These results suggest that Ob-Ra is functional in our culture system and that this leptin receptor isoform plays a role in the regulation of steroidogenesis in human granulosa cells.

Leptin receptors are structurally similar to the class I cytokine receptor family [33]. In humans, the leptin receptor (OB-R) is produced in several alternatively spliced forms, designated OB-Ra, OB-Rb, OB-Rc, OB-Re [34]. Each of these isoforms has an extracellular domain and a transmembrane domain in common, with a variable intracellular domain that is characteristic for each of the isoforms. Based on the variable intracellular domain the isoforms are classified as short (OB-Ra), long (OB-Rb) and secreted (OB-Re) leptin receptor. Other than the classical JAK/STAT signaling pathways [35], leptin may act through OB-Ra or OB-Rb to trigger the MAPK cascade in two different ways, i.e. via tyrosine phosphorylation of JAK2 receptor-associated activation or independently of receptor phosphorylaton [17,36]. Although the molecules that are involved in transmitting the leptin signal have not been completely elucidated, activated MEKs (MAPK/ERK kinases) phosphorylate ERKs, leading finally to the expression of specific target genes, such as *c-fos *and *egr-1*, that participate in cell proliferation and differentiation [37]. The other two members of the MAP kinase family, p38 MAP kinase and NH2-terminal c-Jun kinase/stress-activated protein kinase (JNK) have also been shown to be activated by leptin [38,39].

Many reports have demonstrated the involvement of the MAPK pathway in steroidogenesis in granulosa cells. Activation of the MAPK cascade by gonadotropins mediates down-regulation of steroidogenesis via attenuation of StAR expression [40]. EGF (epidermal growth factor) stimulates steroidogenesis of granulosa cells through activation of the ERK-related MAP kinase pathway [41]. PGF2α (prostaglandin F2α)-induced MAPK activation is associated with modulation of progesterone production [42]. In the present study, we demonstrated that leptin is capable of inducing the phosphorylation of ERK, phosphorylation of p38 and phosphorylation of JNK. Interestingly, only the ERK and p38 inhibitors were able to reverse leptin mediated inhibition of StAR protein expression and progesterone production in granulosa cells. This suggests that these two MAPK cascades are involved in leptin mediated inhibition of steroidogenesis in granulosa cells.

## Conclusion

In conclusion, we demonstrate that the StAR protein participates in the physiological inhibition of granulosa cells function by leptin. This leptin-dependent fine tuning of ovarian function could be of clinical relevance in obesity and related disorders as well as in the pathogenesis of infertility.

## Competing interests

The authors declare that they have no competing interests.

## Authors' contributions

QL and SLP designed the study and performed the experiments and participated in discussion of the results and drafted the manuscript. BHY, LC and PCKL were responsible for supervision of this work. PCKL was responsible for the conception, design, discussion of the results, drafting and critical revision of the manuscript. All authors read and approved the final manuscript.
